# Di-μ-ethano­lato-bis­[diethano­lato(2-methyl­quinolin-8-olato)titanium(IV)]

**DOI:** 10.1107/S1600536809045796

**Published:** 2009-11-07

**Authors:** Yousef Fazaeli, Ezzatollah Najafi, Mostafa M. Amini, Hamid Reza Khavasi

**Affiliations:** a Department of Chemistry, Shahid Beheshti University, G. C., Evin, Tehran 1983963113, Iran

## Abstract

In the centrosymmetric dinuclear title compound, [Ti_2_(C_10_H_8_NO)_2_(C_2_H_5_O)_6_], the Ti atom is bonded to an *N*,*O*-bidentate quinolin-8-olate ligand, two terminal ethano­late anions and two bridging ethano­late anions in a distorted TiNO_5_ octa­hedral geometry. An intra­molecular C—H⋯O hydrogen bond occurs; in the crystal, inter­molecular C—H⋯O inter­actions help to establish the packing.

## Related literature

For Ti^IV^–8-hydroxy­quinolinates, see: Amini *et al.* (2004 ▶); Birdet *et al.* (1973 ▶); Studd & Swallow (1968 ▶). For a related structure, see: Faza­eli *et al.* (2008 ▶).
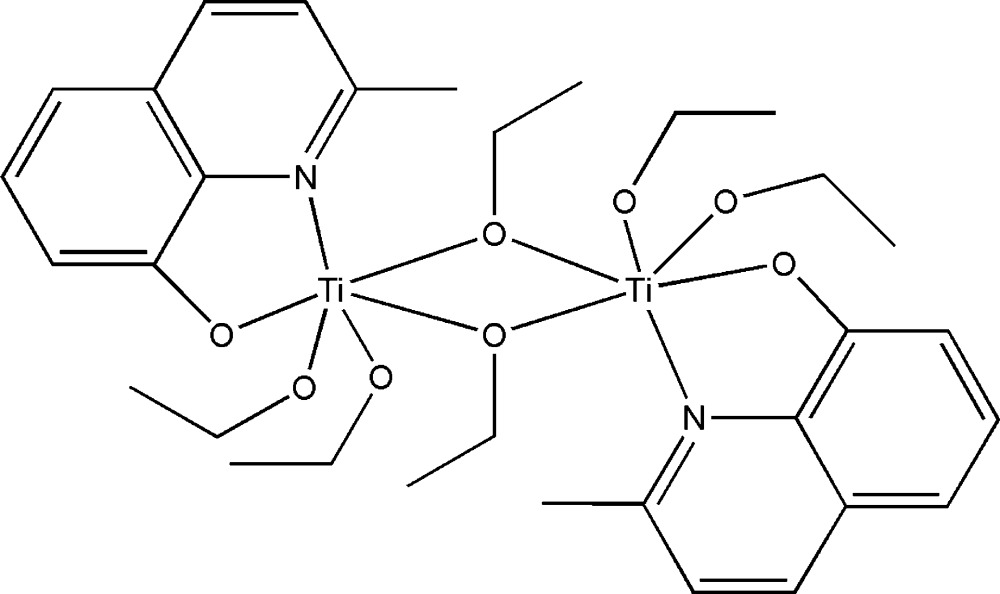


## Experimental

### 

#### Crystal data

[Ti_2_(C_10_H_8_NO)_2_(C_2_H_5_O)_6_]
*M* *_r_* = 682.51
Monoclinic, 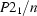

*a* = 9.0497 (18) Å
*b* = 13.086 (3) Å
*c* = 14.189 (3) Å
β = 95.21 (3)°
*V* = 1673.4 (6) Å^3^
*Z* = 2
Mo *K*α radiation
μ = 0.53 mm^−1^
*T* = 120 K
0.45 × 0.28 × 0.23 mm

#### Data collection

Stoe IPDS II diffractometer
Absorption correction: numerical (*X-SHAPE*; Stoe & Cie, 2005 ▶) *T*_min_ = 0.686, *T*_max_ = 0.905
12962 measured reflections
4503 independent reflections
3540 reflections with *I* > 2σ(*I*)
*R*_int_ = 0.099

#### Refinement

*R*[*F* ^2^ > 2σ(*F* ^2^)] = 0.097
*wR*(*F* ^2^) = 0.197
*S* = 1.14
4503 reflections
203 parameters
H-atom parameters constrained
Δρ_max_ = 1.26 e Å^−3^
Δρ_min_ = −1.14 e Å^−3^

### 

Data collection: *X-AREA* (Stoe & Cie, 2005 ▶); cell refinement: *X-AREA*; data reduction: *X-RED* (Stoe & Cie, 2005 ▶); program(s) used to solve structure: *SHELXS97* (Sheldrick, 2008 ▶); program(s) used to refine structure: *SHELXL97* (Sheldrick, 2008 ▶); molecular graphics: *ORTEP-3* (Farrugia, 1997 ▶); software used to prepare material for publication: *WinGX* (Farrugia, 1999 ▶).

## Supplementary Material

Crystal structure: contains datablocks I, global. DOI: 10.1107/S1600536809045796/hb5165sup1.cif

Structure factors: contains datablocks I. DOI: 10.1107/S1600536809045796/hb5165Isup2.hkl

Additional supplementary materials:  crystallographic information; 3D view; checkCIF report

## Figures and Tables

**Table 1:** Selected bond lengths (Å)

Ti1—N1	2.387 (3)
Ti1—O1	1.950 (3)
Ti1—O2	1.808 (3)
Ti1—O3	1.817 (3)
Ti1—O4^i^	2.008 (3)
Ti1—O4	2.061 (2)

Symmetry code: (i) 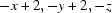.

**Table 2:** Hydrogen-bond geometry (Å, °)

*D*—H⋯*A*	*D*—H	H⋯*A*	*D*⋯*A*	*D*—H⋯*A*
C15—H15*A*⋯O1	0.97	2.46	3.061 (5)	120
C1—H1*C*⋯O3^i^	0.96	2.38	3.292 (5)	159
C3—H3⋯O1^ii^	0.93	2.41	3.310 (5)	163

Symmetry codes: (i) 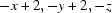; (ii) 
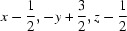
.

**Fig. 1.**
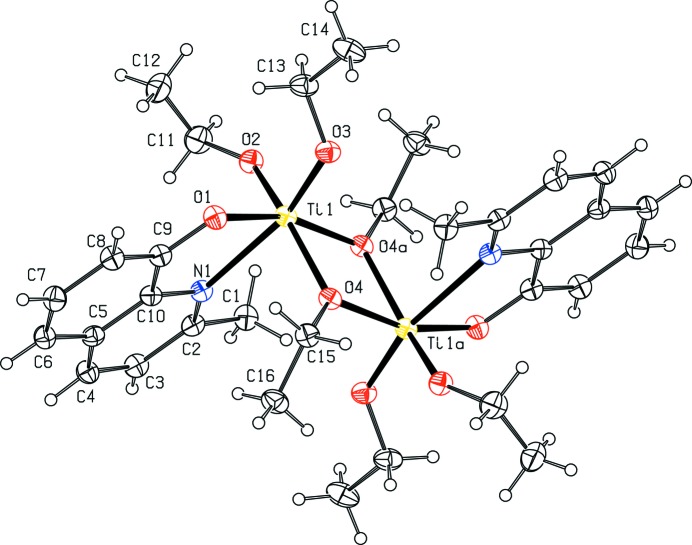
The molecular structure of (I) with displacement ellipsoids drawn at the 30% probability level. Atoms with the suffix a are generated by (2–x, 2–y, –z).

## supplementary crystallographic information

### Comment

8-Hydroxyquinoline and its derivatives are reagents for the gravimetric analysis of metal ions and the crystal structures of a large number of metal 8-hydroxyquinolinates have been documented. However, for Ti^IV^, only three 8-hydroxyquinolinates are known (Amini *et al.*, 2004), (Birdet *et al.*, 1973; Studd & Swallow, 1968). Recently, we have reported the structure of 2-methyl-8-hydroxyquinoline (Fazaeli *et al.*, 2008). In continuation our work in preparation of 8-hydroxyquinoline derivatives of transition metal elements from corresponding alkoxides, here we report synthesis and characterization of the title compound, (I).

This molecule lie across crystallographic inversion centre and the assymetric unit therefore contains one-half of a molecule. The 8-hydroxy-2-methylquinolinate anion chelates to Ti in the triethanolate derivative; however, the coordination number is raised to six as two of the four ethanolate groups are bridging (Table 1) (Fig. 1).

### Experimental

Manipulations were carried out under nitrogen, using standard Schlenk techniques. Titanium^IV^ tetraethoxide was prepared from titanium^IV^ tetrapropoxide (Fluka) and dry ethanol by the alkoxide exchange method and it was puried by vacuum distillation. 8-Hydroxyquinoline (1.6 g, 10 mmol) was added to the reagent (2.28 g, 10 mmol) in toluene (10 ml). The mixture was stirred for a day and the solvent then removed under reduced pressure to furnish a yellow solid. The solid was crystallized from a dichloromethane n-hexane mixture to give yellow prisms of (I).

### Refinement

All H atoms were positioned geometrically, with C—H = 0.93 Å, 0.96Å and 0.97Å for aromatic, methyl and CH_2_ hydrogen atoms respectively and constrained to ride on their parent atoms, with *U*_iso_(H) = 1.2*U*_eq_(C).

### Crystal data

**Table:**

[Ti_2_(C_10_H_8_NO)_2_(C_2_H_5_O)_6_]	*F*(000) = 720
*M**_r_* = 682.51	*D*_x_ = 1.354 Mg m^−^^3^
Monoclinic, *P*2_1_/*n*	Mo *K*α radiation, λ = 0.71073 Å
Hall symbol: -P 2yn	Cell parameters from 1633 reflections
*a* = 9.0497 (18) Å	θ = 2.1–29.2°
*b* = 13.086 (3) Å	µ = 0.53 mm^−^^1^
*c* = 14.189 (3) Å	*T* = 120 K
β = 95.21 (3)°	Prism, yellow
*V* = 1673.4 (6) Å^3^	0.45 × 0.28 × 0.23 mm
*Z* = 2	

### Data collection

**Table:**

Stoe IPDS II diffractometer	4503 independent reflections
Radiation source: fine-focus sealed tube	3540 reflections with *I* > 2σ(*I*)
graphite	*R*_int_ = 0.099
Detector resolution: 0.15 mm pixels mm^-1^	θ_max_ = 29.2°, θ_min_ = 2.1°
rotation method scans	*h* = −12→12
Absorption correction: numerical (*X-SHAPE*; Stoe & Cie, 2005)	*k* = −17→17
*T*_min_ = 0.686, *T*_max_ = 0.905	*l* = −19→15
12962 measured reflections	

### Refinement

**Table:**

Refinement on *F*^2^	Primary atom site location: structure-invariant direct methods
Least-squares matrix: full	Secondary atom site location: difference Fourier map
*R*[*F*^2^ > 2σ(*F*^2^)] = 0.097	Hydrogen site location: inferred from neighbouring sites
*wR*(*F*^2^) = 0.197	H-atom parameters constrained
*S* = 1.14	*w* = 1/[σ^2^(*F*_o_^2^) + (0.1579*P*)^2^ + 0.3709*P*] where *P* = (*F*_o_^2^ + 2*F*_c_^2^)/3
4503 reflections	(Δ/σ)_max_ < 0.001
203 parameters	Δρ_max_ = 1.26 e Å^−^^3^
0 restraints	Δρ_min_ = −1.14 e Å^−^^3^

### Special details

**Table:**

Geometry. All e.s.d.'s (except the e.s.d. in the dihedral angle between two l.s. planes) are estimated using the full covariance matrix. The cell e.s.d.'s are taken into account individually in the estimation of e.s.d.'s in distances, angles and torsion angles; correlations between e.s.d.'s in cell parameters are only used when they are defined by crystal symmetry. An approximate (isotropic) treatment of cell e.s.d.'s is used for estimating e.s.d.'s involving l.s. planes.
Refinement. Refinement of *F*^2^ against ALL reflections. The weighted *R*-factor *wR* and goodness of fit *S* are based on *F*^2^, conventional *R*-factors *R* are based on *F*, with *F* set to zero for negative *F*^2^. The threshold expression of *F*^2^ > σ(*F*^2^) is used only for calculating *R*-factors(gt) *etc*. and is not relevant to the choice of reflections for refinement. *R*-factors based on *F*^2^ are statistically about twice as large as those based on *F*, and *R*- factors based on ALL data will be even larger.

### Fractional atomic coordinates and isotropic or equivalent isotropic displacement parameters (Å^2^)

**Table:**

	*x*	*y*	*z*	*U*_iso_*/*U*_eq_	
C1	0.7281 (4)	0.8994 (3)	−0.1775 (3)	0.0353 (7)	
H1A	0.6738	0.9058	−0.1227	0.053*	
H1B	0.6598	0.8914	−0.2329	0.053*	
H1C	0.7869	0.9596	−0.1839	0.053*	
C2	0.8274 (3)	0.8079 (3)	−0.1667 (2)	0.0306 (7)	
C3	0.8266 (4)	0.7345 (3)	−0.2396 (3)	0.0354 (7)	
H3	0.7650	0.7436	−0.2950	0.042*	
C4	0.9155 (4)	0.6503 (3)	−0.2294 (3)	0.0349 (7)	
H4	0.9165	0.6032	−0.2784	0.042*	
C5	1.0064 (4)	0.6351 (3)	−0.1439 (2)	0.0310 (6)	
C6	1.0989 (4)	0.5492 (3)	−0.1246 (3)	0.0356 (7)	
H6	1.1049	0.4985	−0.1700	0.043*	
C7	1.1805 (4)	0.5410 (3)	−0.0376 (3)	0.0380 (8)	
H7	1.2414	0.4844	−0.0252	0.046*	
C8	1.1734 (4)	0.6166 (3)	0.0329 (3)	0.0349 (7)	
H8	1.2298	0.6094	0.0907	0.042*	
C9	1.0831 (4)	0.7011 (3)	0.0164 (2)	0.0305 (7)	
C10	0.9996 (3)	0.7110 (3)	−0.0735 (2)	0.0284 (6)	
C11	0.6511 (5)	0.7812 (4)	0.0572 (4)	0.0516 (11)	
H11A	0.6560	0.7490	−0.0040	0.062*	
H11B	0.5557	0.8149	0.0573	0.062*	
C12	0.6658 (9)	0.7025 (4)	0.1327 (6)	0.081 (2)	
H12A	0.7618	0.6710	0.1340	0.122*	
H12B	0.5903	0.6515	0.1202	0.122*	
H12C	0.6549	0.7340	0.1928	0.122*	
C13	0.9606 (5)	0.8822 (4)	0.2620 (3)	0.0431 (9)	
H13A	0.9889	0.8109	0.2585	0.052*	
H13B	0.8540	0.8853	0.2650	0.052*	
C14	1.0373 (5)	0.9294 (4)	0.3496 (3)	0.0512 (11)	
H14A	1.1427	0.9274	0.3462	0.077*	
H14B	1.0125	0.8918	0.4042	0.077*	
H14C	1.0057	0.9990	0.3547	0.077*	
C15	1.2662 (4)	0.9251 (3)	−0.0154 (3)	0.0347 (7)	
H15A	1.2825	0.8655	0.0249	0.042*	
H15B	1.3421	0.9750	0.0042	0.042*	
C16	1.2818 (4)	0.8944 (3)	−0.1167 (3)	0.0444 (9)	
H16A	1.2054	0.8464	−0.1371	0.067*	
H16B	1.3772	0.8636	−0.1210	0.067*	
H16C	1.2728	0.9539	−0.1565	0.067*	
N1	0.9147 (3)	0.7980 (2)	−0.0847 (2)	0.0284 (6)	
O1	1.0681 (3)	0.7744 (2)	0.07988 (17)	0.0338 (5)	
O2	0.7640 (3)	0.8529 (2)	0.07192 (19)	0.0356 (6)	
O3	0.9989 (3)	0.9345 (2)	0.17872 (19)	0.0364 (6)	
O4	1.1230 (2)	0.96763 (19)	−0.00251 (17)	0.0302 (5)	
Ti1	0.94929 (6)	0.89765 (5)	0.05650 (4)	0.0280 (2)	

### Atomic displacement parameters (Å^2^)

**Table:**

	*U*^11^	*U*^22^	*U*^33^	*U*^12^	*U*^13^	*U*^23^
C1	0.0283 (15)	0.0410 (18)	0.0341 (17)	0.0002 (13)	−0.0110 (13)	0.0000 (13)
C2	0.0241 (13)	0.0379 (16)	0.0286 (15)	−0.0053 (12)	−0.0048 (11)	0.0032 (12)
C3	0.0296 (15)	0.0430 (18)	0.0320 (17)	−0.0047 (13)	−0.0059 (12)	0.0010 (14)
C4	0.0361 (16)	0.0386 (18)	0.0288 (16)	−0.0035 (14)	−0.0032 (13)	−0.0046 (13)
C5	0.0260 (13)	0.0359 (16)	0.0312 (16)	−0.0044 (12)	0.0024 (11)	0.0014 (13)
C6	0.0354 (16)	0.0327 (16)	0.0387 (18)	0.0010 (13)	0.0034 (14)	−0.0022 (14)
C7	0.0332 (16)	0.0360 (17)	0.044 (2)	0.0052 (14)	0.0013 (14)	0.0007 (15)
C8	0.0311 (15)	0.0390 (18)	0.0331 (17)	0.0036 (13)	−0.0055 (13)	0.0010 (13)
C9	0.0254 (13)	0.0358 (17)	0.0293 (16)	−0.0001 (12)	−0.0031 (11)	0.0016 (12)
C10	0.0237 (13)	0.0350 (16)	0.0258 (14)	−0.0027 (12)	−0.0007 (11)	−0.0008 (12)
C11	0.0382 (19)	0.061 (3)	0.056 (3)	−0.0186 (19)	0.0044 (18)	−0.009 (2)
C12	0.112 (5)	0.039 (3)	0.101 (5)	−0.006 (3)	0.055 (4)	0.002 (3)
C13	0.0396 (18)	0.060 (2)	0.0292 (17)	−0.0043 (17)	0.0005 (14)	0.0001 (16)
C14	0.045 (2)	0.076 (3)	0.0309 (19)	0.013 (2)	−0.0030 (16)	−0.0061 (19)
C15	0.0218 (13)	0.0404 (18)	0.0420 (19)	0.0031 (12)	0.0028 (12)	−0.0011 (14)
C16	0.0322 (17)	0.056 (2)	0.046 (2)	−0.0024 (16)	0.0101 (16)	−0.0103 (18)
N1	0.0224 (11)	0.0346 (14)	0.0269 (13)	−0.0025 (10)	−0.0045 (10)	0.0011 (10)
O1	0.0329 (11)	0.0380 (13)	0.0287 (12)	0.0023 (10)	−0.0073 (9)	−0.0014 (9)
O2	0.0270 (11)	0.0408 (14)	0.0385 (13)	−0.0043 (10)	0.0009 (9)	−0.0014 (10)
O3	0.0329 (12)	0.0463 (15)	0.0295 (12)	−0.0052 (10)	0.0000 (9)	−0.0013 (10)
O4	0.0217 (9)	0.0372 (12)	0.0313 (12)	0.0014 (9)	0.0008 (8)	−0.0026 (9)
Ti1	0.0224 (3)	0.0344 (4)	0.0264 (3)	−0.0008 (2)	−0.0020 (2)	−0.0018 (2)

### Geometric parameters (Å, °)

**Table:**

C1—C2	1.496 (5)	C12—H12A	0.9600
C1—H1A	0.9600	C12—H12B	0.9600
C1—H1B	0.9600	C12—H12C	0.9600
C1—H1C	0.9600	C13—O3	1.435 (5)
C2—N1	1.352 (4)	C13—C14	1.501 (6)
C2—C3	1.411 (5)	C13—H13A	0.9700
C3—C4	1.364 (5)	C13—H13B	0.9700
C3—H3	0.9300	C14—H14A	0.9600
C4—C5	1.417 (5)	C14—H14B	0.9600
C4—H4	0.9300	C14—H14C	0.9600
C5—C6	1.414 (5)	C15—O4	1.438 (4)
C5—C10	1.414 (5)	C15—C16	1.512 (6)
C6—C7	1.384 (5)	C15—H15A	0.9700
C6—H6	0.9300	C15—H15B	0.9700
C7—C8	1.412 (5)	C16—H16A	0.9600
C7—H7	0.9300	C16—H16B	0.9600
C8—C9	1.383 (5)	C16—H16C	0.9600
C8—H8	0.9300	Ti1—N1	2.387 (3)
C9—O1	1.331 (4)	Ti1—O1	1.950 (3)
C9—C10	1.428 (4)	Ti1—O2	1.808 (3)
C10—N1	1.375 (4)	Ti1—O3	1.817 (3)
C11—O2	1.389 (5)	Ti1—O4^i^	2.008 (3)
C11—C12	1.483 (9)	Ti1—O4	2.061 (2)
C11—H11A	0.9700	Ti1—Ti1^i^	3.2948 (13)
C11—H11B	0.9700		
			
C2—C1—H1A	109.5	C14—C13—H13A	109.4
C2—C1—H1B	109.5	O3—C13—H13B	109.4
H1A—C1—H1B	109.5	C14—C13—H13B	109.4
C2—C1—H1C	109.5	H13A—C13—H13B	108.0
H1A—C1—H1C	109.5	C13—C14—H14A	109.5
H1B—C1—H1C	109.5	C13—C14—H14B	109.5
N1—C2—C3	121.9 (3)	H14A—C14—H14B	109.5
N1—C2—C1	117.6 (3)	C13—C14—H14C	109.5
C3—C2—C1	120.4 (3)	H14A—C14—H14C	109.5
C4—C3—C2	120.6 (3)	H14B—C14—H14C	109.5
C4—C3—H3	119.7	O4—C15—C16	112.8 (3)
C2—C3—H3	119.7	O4—C15—H15A	109.0
C3—C4—C5	119.6 (3)	C16—C15—H15A	109.0
C3—C4—H4	120.2	O4—C15—H15B	109.0
C5—C4—H4	120.2	C16—C15—H15B	109.0
C6—C5—C10	119.0 (3)	H15A—C15—H15B	107.8
C6—C5—C4	124.4 (3)	C15—C16—H16A	109.5
C10—C5—C4	116.6 (3)	C15—C16—H16B	109.5
C7—C6—C5	119.5 (3)	H16A—C16—H16B	109.5
C7—C6—H6	120.2	C15—C16—H16C	109.5
C5—C6—H6	120.2	H16A—C16—H16C	109.5
C6—C7—C8	121.5 (3)	H16B—C16—H16C	109.5
C6—C7—H7	119.2	C2—N1—C10	117.1 (3)
C8—C7—H7	119.2	C2—N1—Ti1	133.8 (2)
C9—C8—C7	120.3 (3)	C10—N1—Ti1	109.0 (2)
C9—C8—H8	119.9	C9—O1—Ti1	124.7 (2)
C7—C8—H8	119.9	C11—O2—Ti1	151.4 (3)
O1—C9—C8	123.8 (3)	C13—O3—Ti1	127.0 (2)
O1—C9—C10	117.4 (3)	C15—O4—Ti1^i^	123.9 (2)
C8—C9—C10	118.7 (3)	C15—O4—Ti1	127.5 (2)
N1—C10—C5	124.0 (3)	Ti1^i^—O4—Ti1	108.13 (10)
N1—C10—C9	115.0 (3)	O2—Ti1—O3	97.01 (12)
C5—C10—C9	120.9 (3)	O2—Ti1—O1	102.40 (12)
O2—C11—C12	110.1 (5)	O3—Ti1—O1	88.43 (12)
O2—C11—H11A	109.6	O2—Ti1—O4^i^	93.27 (11)
C12—C11—H11A	109.6	O3—Ti1—O4^i^	100.06 (12)
O2—C11—H11B	109.6	O1—Ti1—O4^i^	161.18 (11)
C12—C11—H11B	109.6	O2—Ti1—O4	160.52 (12)
H11A—C11—H11B	108.2	O3—Ti1—O4	97.94 (11)
C11—C12—H12A	109.5	O1—Ti1—O4	90.44 (10)
C11—C12—H12B	109.5	O4^i^—Ti1—O4	71.87 (10)
H12A—C12—H12B	109.5	O2—Ti1—N1	82.66 (11)
C11—C12—H12C	109.5	O3—Ti1—N1	161.53 (12)
H12A—C12—H12C	109.5	O1—Ti1—N1	73.70 (10)
H12B—C12—H12C	109.5	O4^i^—Ti1—N1	98.39 (10)
O3—C13—C14	111.0 (4)	O4—Ti1—N1	87.05 (10)
O3—C13—H13A	109.4		
			
N1—C2—C3—C4	−0.2 (5)	C11—O2—Ti1—N1	27.3 (6)
C1—C2—C3—C4	−179.1 (3)	C11—O2—Ti1—Ti1^i^	115.5 (6)
C2—C3—C4—C5	1.7 (5)	C13—O3—Ti1—O2	38.1 (3)
C3—C4—C5—C6	177.8 (3)	C13—O3—Ti1—O1	−64.2 (3)
C3—C4—C5—C10	−0.7 (5)	C13—O3—Ti1—O4^i^	132.7 (3)
C10—C5—C6—C7	0.1 (5)	C13—O3—Ti1—O4	−154.4 (3)
C4—C5—C6—C7	−178.5 (4)	C13—O3—Ti1—N1	−49.7 (5)
C5—C6—C7—C8	0.2 (6)	C13—O3—Ti1—Ti1^i^	169.8 (3)
C6—C7—C8—C9	0.3 (6)	C9—O1—Ti1—O2	81.3 (3)
C7—C8—C9—O1	178.5 (3)	C9—O1—Ti1—O3	178.2 (3)
C7—C8—C9—C10	−1.0 (5)	C9—O1—Ti1—O4^i^	−64.3 (4)
C6—C5—C10—N1	179.4 (3)	C9—O1—Ti1—O4	−83.9 (3)
C4—C5—C10—N1	−1.9 (5)	C9—O1—Ti1—N1	2.9 (2)
C6—C5—C10—C9	−0.8 (5)	C9—O1—Ti1—Ti1^i^	−79.3 (3)
C4—C5—C10—C9	177.9 (3)	C15—O4—Ti1—O2	−130.7 (4)
O1—C9—C10—N1	1.5 (4)	Ti1^i^—O4—Ti1—O2	41.8 (4)
C8—C9—C10—N1	−178.9 (3)	C15—O4—Ti1—O3	89.5 (3)
O1—C9—C10—C5	−178.3 (3)	Ti1^i^—O4—Ti1—O3	−98.04 (13)
C8—C9—C10—C5	1.3 (5)	C15—O4—Ti1—O1	1.0 (3)
C3—C2—N1—C10	−2.3 (5)	Ti1^i^—O4—Ti1—O1	173.48 (12)
C1—C2—N1—C10	176.6 (3)	C15—O4—Ti1—O4^i^	−172.5 (3)
C3—C2—N1—Ti1	−178.5 (2)	Ti1^i^—O4—Ti1—O4^i^	0.0
C1—C2—N1—Ti1	0.5 (5)	C15—O4—Ti1—N1	−72.7 (3)
C5—C10—N1—C2	3.4 (5)	Ti1^i^—O4—Ti1—N1	99.83 (12)
C9—C10—N1—C2	−176.4 (3)	C15—O4—Ti1—Ti1^i^	−172.5 (3)
C5—C10—N1—Ti1	−179.5 (3)	C2—N1—Ti1—O2	69.3 (3)
C9—C10—N1—Ti1	0.7 (3)	C10—N1—Ti1—O2	−107.0 (2)
C8—C9—O1—Ti1	176.8 (3)	C2—N1—Ti1—O3	159.5 (3)
C10—C9—O1—Ti1	−3.6 (4)	C10—N1—Ti1—O3	−16.8 (4)
C12—C11—O2—Ti1	88.3 (7)	C2—N1—Ti1—O1	174.6 (3)
C14—C13—O3—Ti1	173.2 (3)	C10—N1—Ti1—O1	−1.8 (2)
C16—C15—O4—Ti1^i^	−67.7 (4)	C2—N1—Ti1—O4^i^	−22.9 (3)
C16—C15—O4—Ti1	103.7 (3)	C10—N1—Ti1—O4^i^	160.7 (2)
C11—O2—Ti1—O3	−134.1 (6)	C2—N1—Ti1—O4	−94.1 (3)
C11—O2—Ti1—O1	−44.2 (6)	C10—N1—Ti1—O4	89.6 (2)
C11—O2—Ti1—O4^i^	125.3 (6)	C2—N1—Ti1—Ti1^i^	−59.2 (3)
C11—O2—Ti1—O4	85.9 (7)	C10—N1—Ti1—Ti1^i^	124.4 (2)

Symmetry codes: (i) −*x*+2, −*y*+2, −*z*.

### Hydrogen-bond geometry (Å, °)

**Table:**

*D*—H···*A*	*D*—H	H···*A*	*D*···*A*	*D*—H···*A*
C15—H15A···O1	0.97	2.46	3.061 (5)	120
C1—H1C···O3^i^	0.96	2.38	3.292 (5)	159
C3—H3···O1^ii^	0.93	2.41	3.310 (5)	163

Symmetry codes: (i) −*x*+2, −*y*+2, −*z*; (ii) *x*−1/2, −*y*+3/2, *z*−1/2.
